# Supplementary material for: Flower consumption, ambient temperature and rainfall modulate drinking behavior in a folivorous-frugivorous arboreal mammal
Source: PLoS One. 2021 Feb 19;16(2):e0236974. doi: 10.1371/journal.pone.0236974 (PMC7894884; doi:10.1371/journal.pone.0236974)
Supplement: S1 File — (DOCX) [file pone.0236974.s001.docx]

Chaves OM, Fortes VB, Hass GP, Azevedo RB, Stoner KE, Bicca-Marques JC. Flower consumption, ambient temperature and rainfall modulate drinking behavior in a folivorous-frugivorous arboreal mammal. PLoS ONE. 2021.


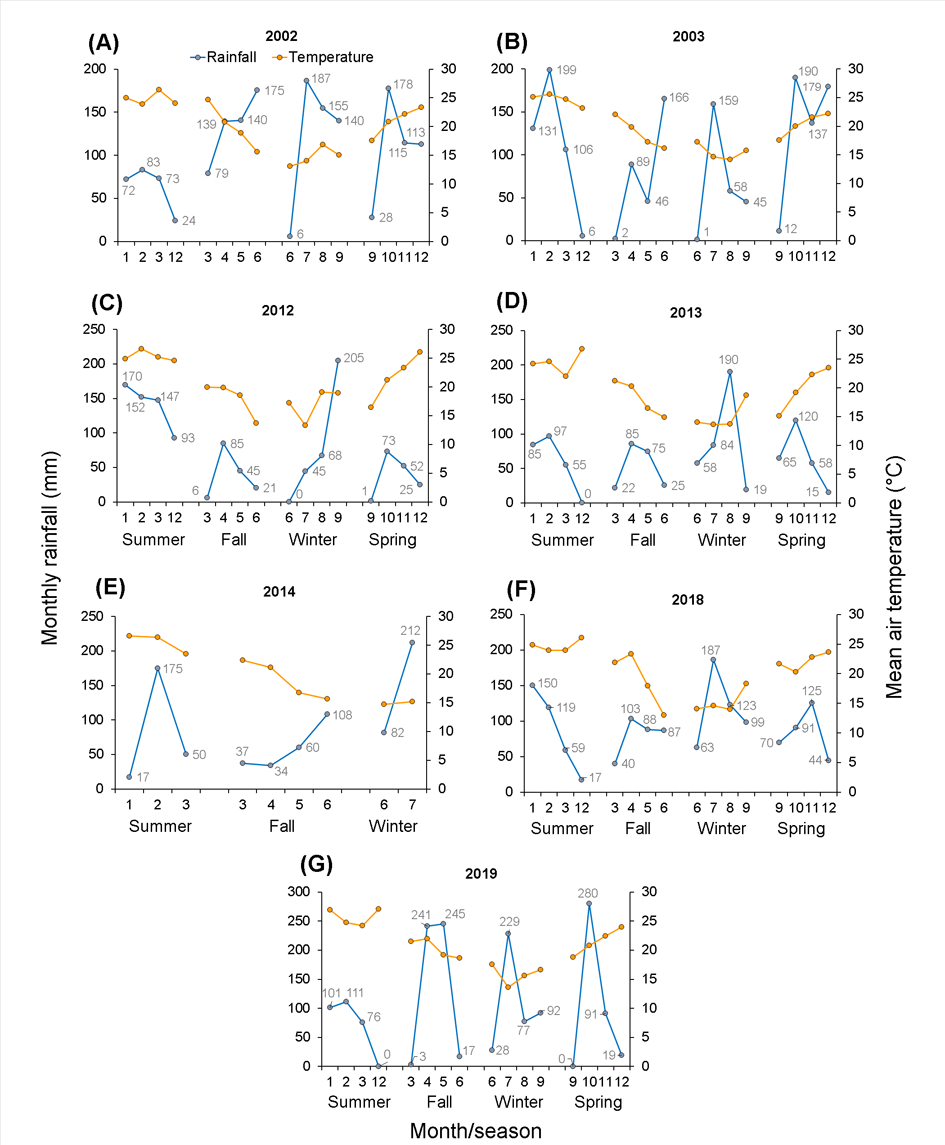
**S1 Fig.** Rainfall (blue line) and mean air temperature (yellow line) in Porto Alegre and Viamão municipallities during the study. Data on air temperature for all study years and rainfall data for 2002, 2003, 2018, and 2019 (A, B, F, and G) come from the Instituto Nacional de Meteorologia do Brasil (INMET, 2019). Rainfall data for the other years were recorded on pluviometers installed in fragments S1, S4 and L1. Numbers on the X-axes are sampling months in each season. Start and end dates of the seasons in the study region (ca. 31ºS), Summer: 21 December-20 March, Fall: 21 March-20 June, Winter: 21 June-22 September, Spring: 22 September-21 December. Grey numbers represent the monthly rainfall in mm.


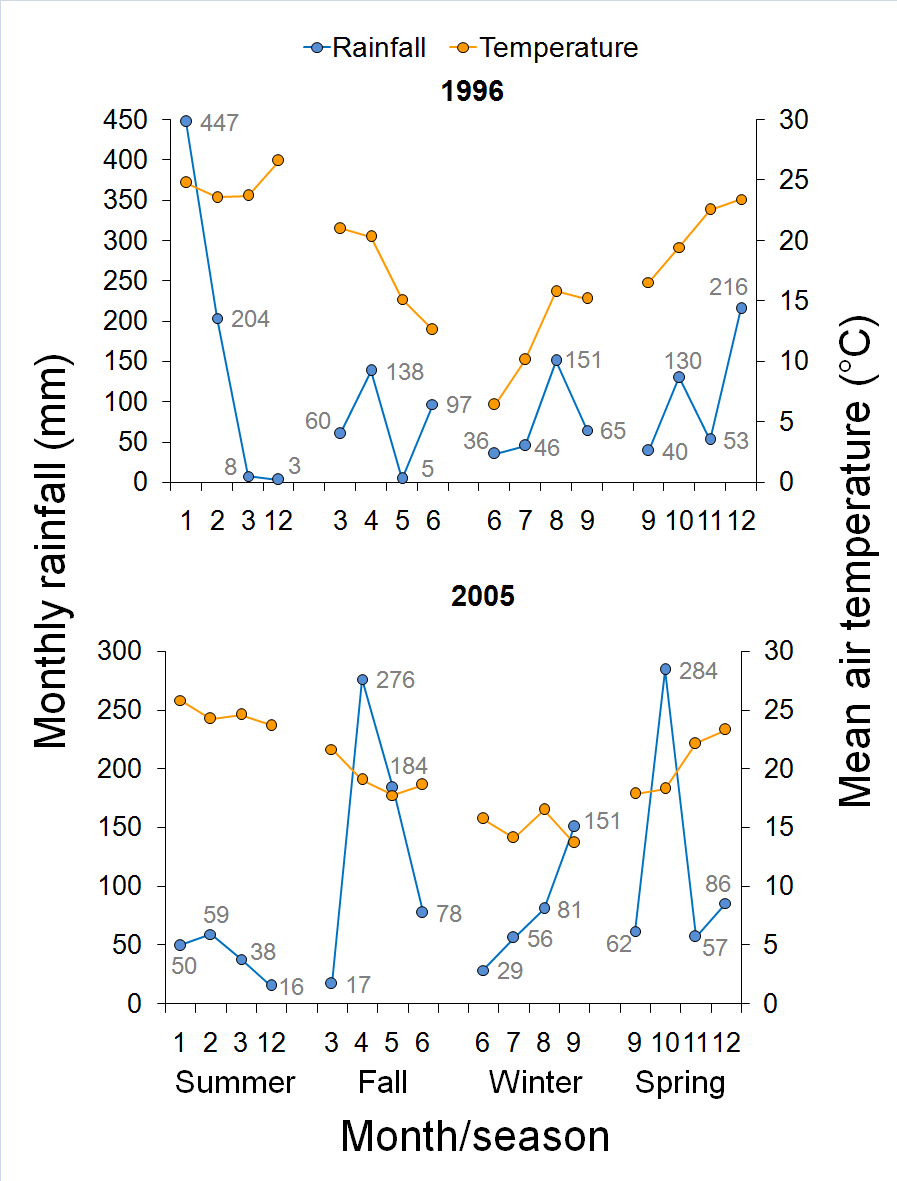


**S2 Fig.** Rainfall (blue) and mean air temperature (yellow) in Santa Maria municipality during the study years of 1996 and 2005. Data for both variables come from the meteorological stations of the Instituto Nacional de Meteorologia do Brasil (INMET, 2019). Start and end dates of the seasons in the study region (ca. 31ºS), Summer: 21 December-20 March, Fall: 21 March-20 June, Winter: 21 June-22 September, Spring: 23 September-20 December. Numbers on the X-axes represent the sampling months in each season. Grey numbers represent the monthly rainfall in mm.


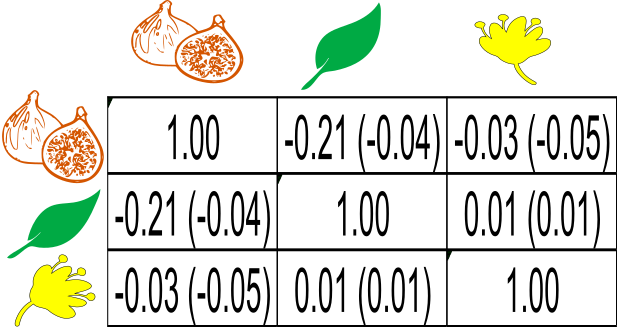


**S3 Fig.** Matrix of Spearman and Pearson (in parentheses) correlation coefficients between the consumption of fruits, leaves and flowers by14 brown howler monkey (*Alouatta guariba clamitans*) groups in the State of Rio Grande do Sul, Brazil.


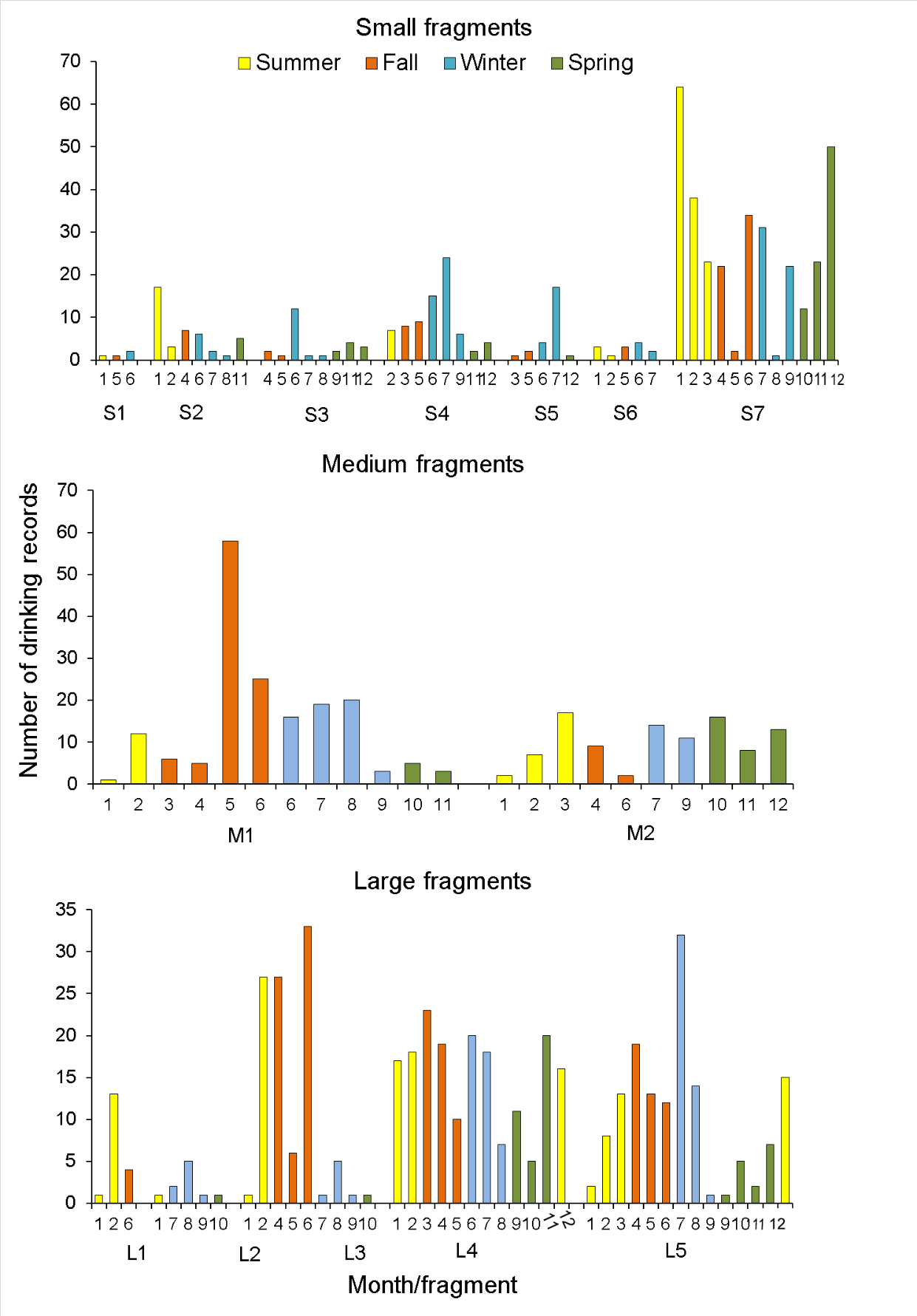


**S4 Fig.** Seasonal distribution of drinking events by 14 brown howler groups inhabiting small, medium, and large Atlantic forest fragments in southern Brazil. Start and end dates of the seasons in the study region (ca. 31ºS), Summer: 21 December-20 March, Fall: 21 March-20 June, Winter: 21 June-22 September, Spring: 23 September-20 December.


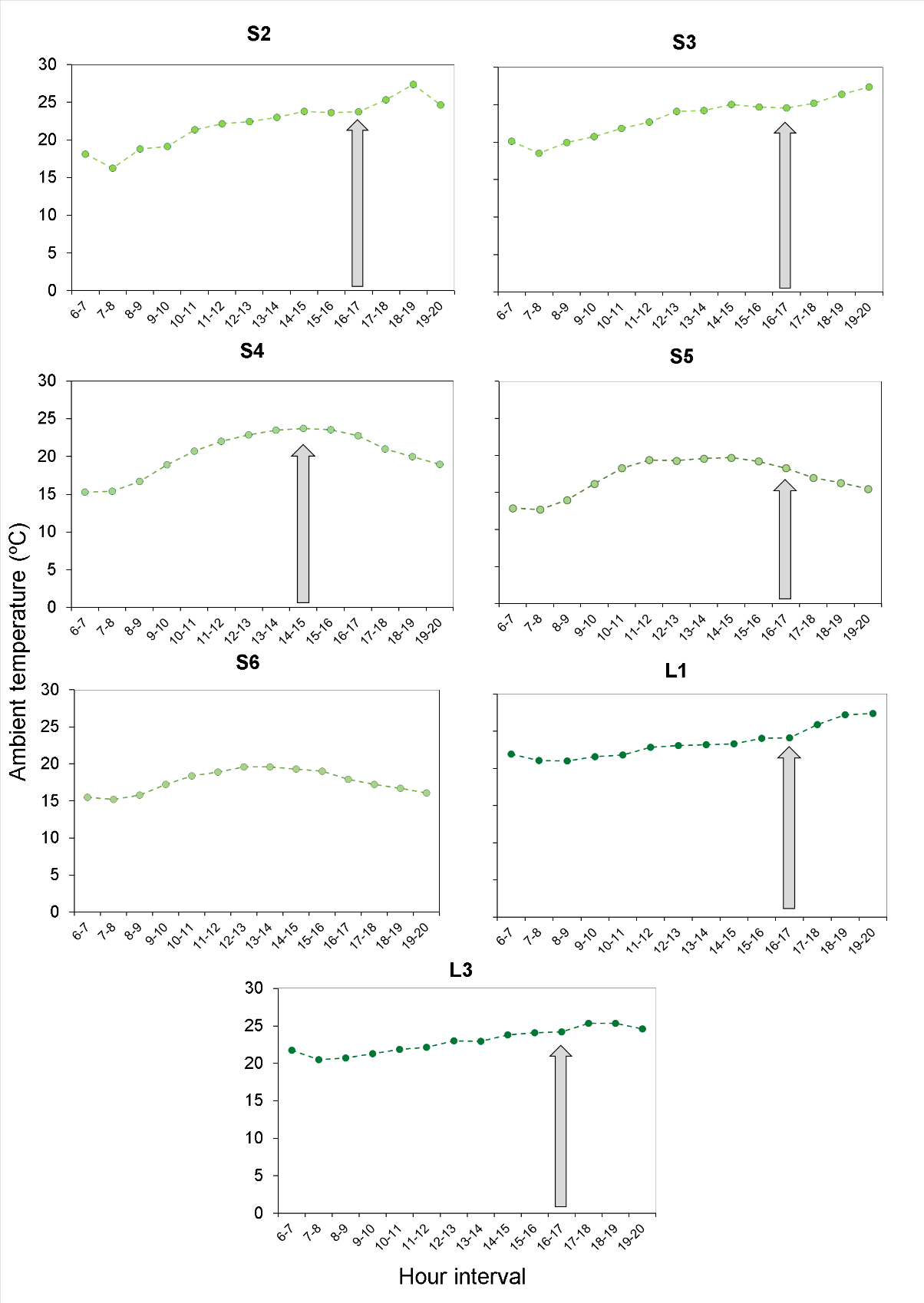


**S5 Fig.** Hourly variation in average ambient temperature of seven Atlantic Forest fragments in Porto Alegre (S2, S3, L1) and Viamäo municipalities (S4, S5, S6, L3), southern Brazil. Temperatures recorded in each study fragment from August 2011 to June 2014 and from June 2018 to July 2019. The gray arrow indicates the hour with the highest number of drinking records by brown howler monkeys in those groups in which drinking records varied significantly during the day (see also Fig. 3).

**References**

INMET. Banco de Dados meteorológicos para ensino e pesquisa: registros metereologicos para Rio Grande do Sul.Instituto Nacional de Metereologia do Brasil, Porto Alegre, 2019. [https://www.inmet.gov.br/ portal/ index.php?r =bdmep/bdmep](https://www.inmet.gov.br/%20portal/%20index.php?r%20=bdmep/bdmep).
